# Characteristics of a Plasticized PVA-Based Polymer Electrolyte Membrane and H^+^ Conductor for an Electrical Double-Layer Capacitor: Structural, Morphological, and Ion Transport Properties

**DOI:** 10.3390/membranes11040296

**Published:** 2021-04-20

**Authors:** Mohamad A. Brza, Shujahadeen B. Aziz, Hazleen Anuar, Saad M. Alshehri, Fathilah Ali, Tansir Ahamad, Jihad M. Hadi

**Affiliations:** 1 Department of Manufacturing and Materials Engineering, Faculty of Engineering, International Islamic University of Malaysia, Kuala Lumpur, Gombak 53100, Malaysia; mohamad.brza@gmail.com (M.A.B.); hazleen@iium.edu.my (H.A.); 2 Advanced Polymeric Materials Research Lab., Department of Physics, College of Science, University of Sulaimani, Qlyasan Street, Sulaimani 46001, Iraq; 3 Department of Civil Engineering, College of Engineering, Komar University of Science and Technology, Sulaimani 46001, Iraq; 4 Department of Chemistry, King Saud University, P.O. Box 2455, Riyadh 11451, Saudi Arabia; alshehri@ksu.edu.sa (S.M.A.); tahamed@ksu.edu.sa (T.A.); 5 Department of Biotechnology Engineering, Kulliyyah of Engineering, International Islamic University Malaysia (IIUM), Jalan Gombak, Kuala Lumpur 53100, Malaysia; fathilah@iium.edu.my; 6 Department of Medical Laboratory of Science, College of Health Sciences, University of Human Development, Kurdistan Regional Government, Sulaimani 4600, Iraq; jihad.chemist@gmail.com

**Keywords:** solid polymer electrolyte, ammonium thiocyanate, PVA polymer, glycerol, electrochemical double-layer capacitor device, electrochemical properties

## Abstract

Poly (vinyl alcohol) (PVA)-based solid polymer electrolytes doped with ammonium thiocyanate (NH_4_SCN) and glycerol were fabricated using a solution casting method. Lithium-based energy storage devices are not environmentally friendly materials, and they are toxic. Thus, proton-conducting materials were used in this work as they are harmless and are smaller than lithium. The interaction between PVA and the electrolyte elements was shown by FTIR analysis. The highest conductivity of 1.82 × 10^−5^ S cm^−1^ was obtained by the highest-conducting plasticized system (PSP_2) at room temperature. The mobility, diffusion coefficient, and number density of anions and cations were found to increase with increasing glycerol. FESEM was used to investigate the influence of glycerol on film morphology. TNM showed that the cations and anions were the main charge carriers. LSV showed that the electrochemical stability window of the PSP_2 system was 1.99 V. The PSP_2 system was applied in the preparation of an electrical double layer capacitor device. The shape of the cyclic voltammetry (CV) curve was nearly rectangular with no Faradaic peaks. From the galvanostatic charge-discharge analysis, the power density, energy density, and specific capacitance values were nearly constant beyond the first cycle at 318.73 W/Kg, 2.06 Wh/Kg, and 18.30 F g^−1^, respectively, for 450 cycles.

## 1. Introduction

There are two main kinds of electrochemical supercapacitors as categorized by the mechanism of energy storage: (1) electrical double-layer capacitor (EDLC) and (2) electrochemical pseudocapacitor (PSc) [1,2]. An EDLC can store charges in the double-layer at the interfaces between the electrolytes and electrode, while the PSc sustains a charge transfer reaction (Faradaic reaction) between the electrolytes and electrode in an appropriate potential window. Activated carbon (AC) electrode materials with a large surface area of 2500 m^2^/g have been used for EDLCs [3]. The large surface area of AC causes more electrosorption of NH_4_^+^ cations and SCN^−^ anions, and this leads to a higher capacitance. Furthermore, AC porosity is imperative in the preparation of EDLCs with higher capacitance.

The use of solid polymer electrolytes (SPEs) as separators in electrochemical energy storage devices, such as supercapacitors and batteries, causes a decrease in reactivity and in leakage issues related to the liquid electrolyte. In addition, SPEs can provide desired shapes, better safety, and good flexibility. However, their low conductivity, poor interfacial stability between electrodes, and poor mechanical strength are still issues for researchers [4,5]. To provide better conductivity of SPEs, numerous methods have been used, such as the addition of ionic liquids and plasticizers and the blending of polymers to form blended and plasticized polymer electrolytes (PEs) [6,7], gel polymer electrolytes [8], and ionic liquid-based PEs.

In this work, a poly (vinyl alcohol) (PVA)-based SPE incorporated with ammonium thiocyanate (NH_4_SCN) and glycerol was successfully prepared, and the plasticized electrolyte was used for the preparation of an EDLC device. Ammonium salts are broadly used in proton-conducting PEs because they are good proton donors [9]. NH_4_SCN salt has a lattice energy of 605 kJ/mol and thus is easily dissociated into cations and anions when it is dissolved in the water solvent, therefore, NH_4_SCN delivers more ammonium ions to the polymer [9]. Hemalatha et al. [9] prepared PVA/amino acid proline doped with different concentrations of NH_4_SCN. The authors showed that the amorphous phase and conductivity increased by adding 0.4 and 0.5 Mwt% NH_4_SCN, while they decreased with 0.6 Mwt% NH_4_SCN. Hema et al. obtained the conductivity of 10^−5^ S cm^−1^ for the system of PVA/NH_4_Cl [10]. Asnawi et al. [11] prepared a PE system of chitosan/dextran-NaTf with different glycerol concentrations. The plasticized electrolyte with the highest conductance had a maximum direct current (DC) ionic conductivity of 6.10 × 10^−5^ S/cm. The author used the electrolyte for application in electrochemical energy storage devices. Dey et al. [12] fabricated polyethylene oxide (PEO)/potassium iodide (KI) PEs. The authors showed the effect of the filler of nanosized ceria (CeO_2_~10 nm) on ionic conductivity in the PE of PEO/KI. They showed that the addition of the nanoparticles (NPs) increased the conductivity by two orders of magnitude. The authors indicated that the ionic conductivity increased with the addition of KI salt. The conductivity was more increased by the incorporation of CeO_2_ NPs. The maximum conductivity of 2.15 × 10^−3^ S cm^−1^ was achieved for 20 wt. % of CeO_2_ doped-PE composite. PVA is biocompatible and biodegradable, and it is non-toxic. Shuhaimi et al. [13] documented that H^+^ ions are the conducting charges, which mobilize in the κ-carrageenan/chitosan/NH_4_NO_3_ system. It has been described that H^+^ ions in the PEO/NH_4_SCN system are the conducting charges [14]. In this research, glycerol was used to improve conductivity. Plasticizers can improve the amorphous structure of the PEs [3]. Pawlicka et al. [15] increased the conductivity from around 10^−8^ to 10^−4^ S cm^−1^ by the addition of glycerol in their electrolytes. Based on an elastic poly (acrylic acid) (PAA)-based gel electrolyte film, eutectic gallium-indium liquid metal (EGILM) anode, and carbon fiber yarn coated by a Pt nanoflower array as the cathode, Liu et al. [16] showed a cable-shaped, highly elastic, soft, discharge-current-controllable liquid metal–air battery with high flexibility, high discharge performance, and low operating temperature. The energy concerns and related environmental problems have made the study of energy fields and electrochemical energy storage devices a hot subject since the beginning of 21st century. Each year, thousands of scientific works on electrochemical energy storage devices are published. Through our research in this area, we intend to create commercially viable polymer electrolyte-based electrochemical energy storage devices. Attaining this aim requires testing of different polymer-based electrolyte systems, and improving various properties of the polymer electrolyte so as to reach the best solution. Using biodegradable polymer-based electrolytes, for example PVA, can have both economic and environmental benefits. Lithium-based electrochemical energy storage devices are not eco-friendly materials and they are poisonous. Instead, proton-conducting species are smaller than lithium and they are harmless. In this work, a proton-conducting PVA-based electrolyte is fabricated for the use in an EDLC device.

## 2. Materials and Methods

### 2.1. Materials

Glycerol with a molecular weight (Mw) of 92.09382 g/mol and PVA with an average Mw of 85,000–124,000 was purchased from Sigma-Aldrich. NH_4_SCN with a Mw of 76.12 g·mol^−1^, *n*-Methyl-2-pyrrolidone with a Mw of 99.13 g·mol^−1^, and carbon black with a Mw of 12.01 g·mol^−1^ were purchased from HmbG chemicals, EMPLURA, and Timcal, respectively (Kuala Lumpur, Malaysia). Activated carbon with a Mw of 12.01 g·mol^−1^ and polyvinylidene fluoride with an average Mw of ~534,000 by GPC were purchased from Magna value.

### 2.2. Electrolyte Preparation

First, 50. wt. % PVA was dissolved in 40 mL distilled water, and it was stirred with a magnetic stirrer at 85 °C for 65 min for the preparation of a PVA solution and was allowed to cool to room temperature (RT). The solutions of PVA were combined with 50 wt. % NH_4_SCN and stirred with a magnetic stirrer at RT until the NH_4_SCN in the solutions was dissolved. Lastly, PVA/NH_4_SCN was plasticized with 30 and 40 wt. % glycerol, and it was coded as PSP_1 and PSP_2, respectively. The elements of the solutions were stirred to prepare a homogeneous solution and then kept at RT in the plastic Petri dishes for drying. For more drying, the samples were kept in a desiccator with blue silica gel before characterization and preparation of the EDLC device.

### 2.3. Characterization Techniques

#### 2.3.1. Fourier Transform Infrared Spectroscopy (FTIR)

To investigate the PSP_1, PSP_2, and PVA films, an FTIR Spectrophotometer (Malvern Panalytical Ltd., Malvern, UK) was used in the wavenumber range 4000 to 450 cm^−1^ using a resolution of 2 cm^−1^.

#### 2.3.2. Field Emission Scanning Electron Microscopy (FESEM)

An Hitachi SU8220 (FEI Quanta 200 FESEM, FEI Company, Hillsboro, OR, USA) was used to conduct FESEM at a magnification of 500× to study the film’s morphology.

#### 2.3.3. Electrochemical Impedance Spectroscopy (EIS)

The impedance data of PSP_1, PSP_2, and PVA films were taken using an EIS (3532-50 LCR HiTESTER (HIOKI), Nagano, Japan) between 50 Hz and 5 MHz. The films were cut into circles with a radius of 1 cm, and the films were inserted between two stainless steel (SS) electrodes by spring pressure. The imaginary and real parts (Z″ and Z′) of the complex impedance (Z*) data were measured by connecting the cell with a computer program.

### 2.4. Electrolyte Characterization

#### Transference Number Measurement (TNM) and Linear Sweep Voltammetry (LSV)

The TNM of ions (*t_ion_*) and TNM of electrons (*t_el_*) were measured. The arrangement of the cell was SS|PSP_2|SS, and it was connected to a V&A Instrument DP3003 digital DC power supply and UNI-T UT803 multimeter. The applied voltage was 0.2 V at RT and the cell was polarized with time.

The electrochemical stability window (ESW) of the PSP_2 was measured using an LSV employing a DY2300 potentiostat at a scan rate of 10 mV s^−1^ and with a potential between 0 and 2.5 V.

### 2.5. Electrode Preparation

The preparation of the activated carbon electrodes was shown in our previous work in detail [3].

### 2.6. EDLC Characterization

**Figure 1:**
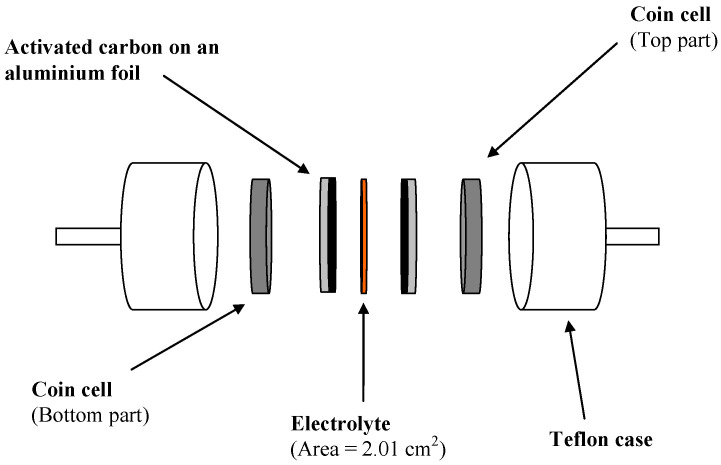
Schematic diagram of the electrical double-layer capacitor (EDLC) setup.

The activated carbon (AC) electrode was cut into circles with an area of 2.01 cm^2^. The PSP_2 was inserted between the electrodes and then placed in a CR2032 coin cell. After that, the CR2032 was placed in a Teflon case as indicated in Figure 1.

## 3. Results and Discussion

### 3.1. FTIR Analysis

**Figure 2:**
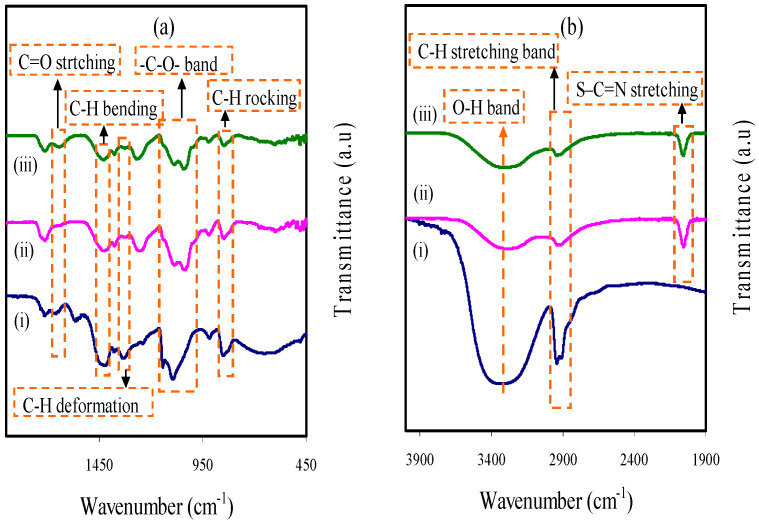
FTIR spectra for (**i**) pure poly (vinyl alcohol) (PVA), (**ii**) PSP_1, and (**iii**) PSP_2 from (**a**) 450 to 1900 cm^−1^ and (**b**) 1900 to 4000 cm^−1^.

The FTIR for PVA/NH_4_SCN/glycerol and PVA are shown in Figure 2a,b. The modifications in the spectral features were seen when the PSP_1 and PSP_2 were compared with the PVA. The O–H stretching is the cause for the broad peak at 3303 cm^−1^ [17,18], while there were a shift and decrease in its intensity in the plasticized PVA due to the interaction between the elements of the electrolyte. Asymmetric stretching of C–H is associated with a peak at 2902 cm^−1^ [17], and its intensity is shifted and decreased in the case of plasticized PVA.

The new intense and strong peak that emerged at 2043 cm^−1^ is related to aromatic S–C = *n* stretching of SCN^−^ of the salt, while its intensity is shifted and falls with increasing glycerol by the interaction of the PVA with the electrolyte elements (see Figure 2b) [9,19]. As one of four H^+^ in the tetrahedral NH_4_^+^ connected to the nitrogen atom is weakly bound, H^+^ transfers to the coordination sites in PVA. The shift in the peak and the new peak in the plasticized PVA shows the interaction between the NH_4_SCN and the PVA [9]. The plasticizer can dissociate more salts to cations and anions and thus more ions are created to interact with the PVA [20].

C=O stretching of the acetate group is related to the absorption band at 1638 cm^−1^ in PVA [21], while it shifted to smaller wavenumbers in the plasticized PVA. The C–H bending of CH_2_ wagging is related to the band at 1412 cm^−1^ in PVA, and C–H deformation is related to the band at 1318 cm^−1^ in PVA (see Figure 2a,) [21]. The intensity of the two peaks was decreased and shifted in the plasticized PVA due to the interaction between the electrolyte elements.

In addition, the –C–O– stretching in PVA is the source for the band at 1080 cm^−1^ [22], which is decreased and shifted in its intensity in PSP_1 and PSP_2, as indicated in Figure 2a. C–H rocking in PVA is the cause for the band at 833 cm^−1^ (see Figure 2a,) [18]. In the plasticized PVA films, the intensity of the peak decreased and shifted, while a 40 wt. % glycerol addition caused a noticeable decrease in the intensity of the peak. These results are shown in Table 1.

### 3.2. Impedance Analysis

**Figure 3:**
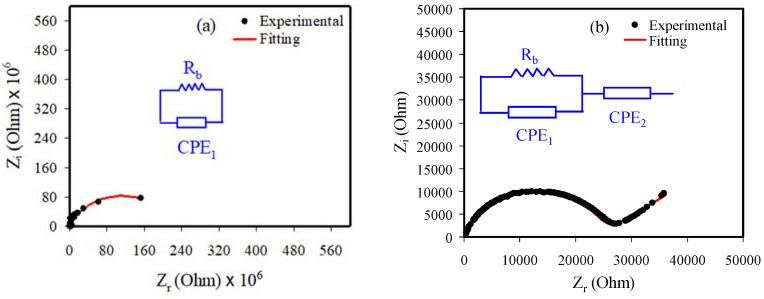

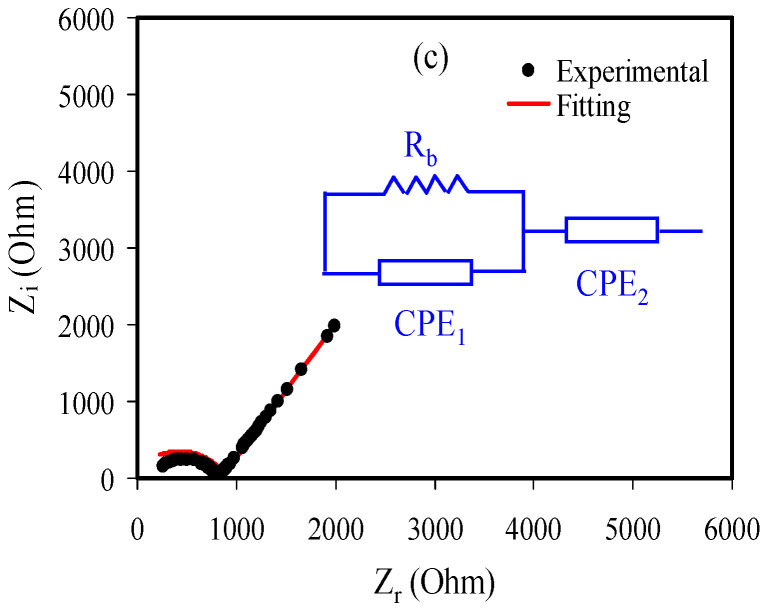
EIS for (**a**) PVA, (**b**) PSP_1, and (**c**) PSP_2at room temperature (RT).

Figure 3a–c shows the Nyquist plot for each film of plasticized PVA and PVA at ambient temperature. The bulk resistance (*R_b_*) is measured using the intersection of the line with a real axis. The *R_b_* reduces as the glycerol is increased to 40 wt. % owing to the increase of the mobility of carriers, and thus the conductivity increases [23].

The model of an electrical equivalent circuit (EEC) is used to fit the EIS data because it is a simple method [24]. The *R_b_* is measured by the intersection between the semicircle and the horizontal axis at the low frequency region. The pure PVA consists of only a semicircle, while with the addition of 30 and 40 wt. % glycerol (PSP_1 and PSP_2), the EIS consists of a tail and one semicircle at the regions of low and high frequencies, respectively as shown in Figure 3a–c. The semicircle is associated with the anions and cations conduction at the bulk of the system [25]. It has been reported that the bulk conductivity is related to the parallel connection of bulk capacitance and *R_b_* of the electrolyte [26]. The spike line is due to the migration of ions at the electrolyte and electrode interfaces [27].

The Nyquist plots for the pure PVA are interpreted as a combination of a constant phase element (CPE_1_) and *R_b_* for the carriers in the electrolytes, while the PSP_1 and PSP_2 systems are interpreted as a combination of *R_b_* and two CPEs (CPE_1_ and CPE_2_), as indicated in the insets of Figure 3a–c [28].

The impedance of *Z_CPE_* is shown as [29,30]:

(1) $$Z_{CPE}=\frac{1}{C\omega^{p}}\left[\cos\left(\frac{\pi p}{2}\right)-i\sin\left(\frac{\pi p}{2}\right)\right]$$

where *ω*, *C*, and *p* are the angular frequency, capacitance of CPE, and deviation of the Cole–Cole plots from the vertical axis, respectively.

The imaginary and real parts (*Z_i_* and *Z_r_*) of the impedance related to the EEC (inset of Figure 3a) are interpreted as:

(2) $$Z_{r}=\frac{R_{b}{}^{2}C_{1}\omega^{p_{1}}\cos\left(\frac{\pi p_{1}}{2}\right)+R_{b}}{2R_{b}C_{1}\omega^{p_{1}}\cos\left(\frac{\pi p_{1}}{2}\right)+R_{b}{}^{2}C_{1}{}^{2}\omega^{2p_{1}}+1}$$

(3) $$Z_{i}=\frac{R_{b}{}^{2}C_{1}\omega^{p_{1}}\sin\left(\frac{\pi p_{1}}{2}\right)}{2R_{b}C_{1}\omega^{{}^{p}}\cos\left(\frac{\pi p_{1}}{2}\right)+R_{b}{}^{2}C_{1}{}^{2}\omega^{2P_{1}}+1}$$

The *Z_r_* and *Z_i_* associated with the EEC as presented in the inset of Figure 3b,c are interpreted as:

(4) $$Z_{r}=\frac{R_{b}{}^{2}C_{1}\omega^{p1}\cos\left(\frac{\pi p_{1}}{2}\right)+R_{b}}{2R_{b}C_{1}\omega^{p1}\cos\left(\frac{\pi p_{1}}{2}\right)+R_{b}{}^{2}C_{1}{}^{2}\omega^{2p1}+1}+\frac{\cos\left(\frac{\pi p_{2}}{2}\right)}{C_{2}\omega^{p2}}$$

(5) $$Z_{i}=\frac{R_{b}{}^{2}C_{1}\omega^{p1}\sin\left(\frac{\pi p_{1}}{2}\right)}{2R_{b}C_{1}\omega^{{}^{p1}}\cos\left(\frac{\pi p_{1}}{2}\right)+R_{b}{}^{2}C_{1}{}^{2}\omega^{2P1}+1}+\frac{\sin\left(\frac{\pi p_{2}}{2}\right)}{C_{2}\omega^{p2}}$$

where *C*_1_ is the bulk capacitance of CPE_1_ and *C*_2_ is the capacitance of CPE_2_ at the interface between the electrode and the electrolyte. The EEC fitting parameters are indicated in Table 2.

By taking *R_b_* and the electrolyte dimensions, the DC conductivity (*σ_dc_*) is measured using the Equation (6) [31,32],

(6) $$\sigma_{dc}=\left(\frac{1}{R_{b}}\right)\times \left(\frac{t}{A}\right)$$

where *A* and *t* are the area of the SS electrodes and thickness of the electrolyte, respectively. The electrolytes’ conductivities are shown in Table 3. Based on the previous study, the conductivity of 10^−5^ S cm^−1^ is reasonable for use in energy storage devices [11,33].

It was previously shown that the incorporation of NH_4_NO_3_ (30 wt. %) into a potato starch/methyl cellulose blend system improved the conductivity to (4.37 ± 0.16) × 10^−5^ S cm^−1^ [34]. In this study with 40 wt. % of glycerol, the DC conductivity increased to 1.82 × 10^−5^ S cm^−1^. Plasticization causes an increase in the dissociation and forms salt-conducting pathways for the transportation of mobile cations and anions, which increase the conductivity [35]. The large dielectric constant of glycerol can decrease the electrostatic force between anions and cations of the salt and thus deliver more mobile ions. The existence of plasticizer can increase the amorphous nature of the PE and thus increase the ionic conductivity [29]. Hamsan et al. [29] documented that the decrease of conductivity by the addition of 50 wt. % glycerol is attributed to the arrangement of self-linkages of plasticizer, causing recrystallization of the salt, which causes a decrease in conductivity.

As the impedance data of the PSP_1 and PSP_2 systems consist of a spike and one semicircle, the number density (*n*), diffusion coefficient (*D*), and mobility (*μ*) of ions are obtained using the relations below [36,37,38]:

The *D* of the plasticized systems is measured using Equation (7).

(7) $$D=\left(\frac{{\left(K_{2}\varepsilon_{o}\varepsilon_{r}A\right)}^{2}}{\tau_{2}}\right)$$

where *ε_r_* and *ε_o_* are the dielectric constant and the permittivity of the vacuum, respectively. *τ_2_* is the reciprocal of *ω* corresponding to the smallest value in *Z_i_*.

The mobility (*µ*) of the plasticized systems is measured using the equation of Nernst–Einstein,

(8) $$\mu =\left(\frac{eD}{K_{b}T}\right)$$

where *T* and *k_b_* refer to the absolute temperature and Boltzmann constant, respectively.

Conductivity is indicated by

(9) $$\sigma_{dc}=ne\mu$$

Hence, the number density of ions (*n*) is measured by Equation (10):

(10) $$n=\left(\frac{\sigma_{dc}K_{b}T\tau_{2}}{{\left(eK_{2}\varepsilon_{o}\varepsilon_{r}A\right)}^{2}}\right)$$

Table 4 lists the transport parameters of ions for the systems.

As seen in Table 4, the *D* increased as the glycerol increased from 30 to 40 wt. %. A similar trend was noted for *μ*, as see in Table 4. The *D* and *μ* increments are related to the development of chain flexibility due to glycerol. The values of *n*, *μ*, and *D* increased with glycerol, which causes to increase in the conductivity, as more glycerol incorporation can dissociate further salts to free ions, and hence raise the *n* of ions [3].

### 3.3. Field Emission Scanning Electron Microscopy (FESEM)

**Figure 4:**
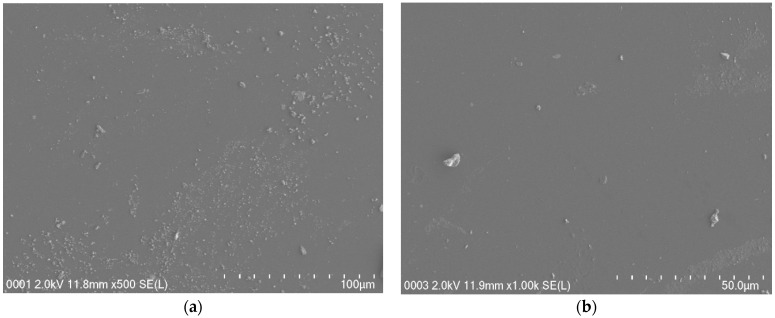
FESEM for (**a**) PSP_1 and (**b**) PSP_2 electrolyte systems at RT.

Field emission scanning electron micrographs were taken at a 500× magnification for plasticized systems to support the EIS, and the micrographs for the systems are shown in Figure 4a,b. The white structures show the protruding salts within the sample’s surface in Figure 4a,b. When there is an addition of 30 wt. % glycerol into the system, a few salts emerged on the sample surface, as shown in Figure 4a. It is seen from the field emission scanning electron micrograph in Figure 4b that protruding salt structures were not obviously seen as the glycerol increased to 40 wt. % (PSP_2 system) compared with that seen for the PSP_1 electrolyte system. The glycerol plasticizer dissociates more salts into anions and cations and thus improves the value of the conductivity [29]. The PSP_2 film has a uniform and smooth surface morphology and has no phase separation. The field emission scanning electron micrographs are in good agreement with the EIS. The white structures decreased in the PSP_2 system and the *R_b_* decreased as shown in the EIS plot, while conductivity increased. It has been reported that the smooth surface morphology is related to the amorphous structure development of the systems [39]. The smooth surface electrolyte can help conduct ions and allow them to migrate easily, and thus increase the DC ionic conductivity [39].

### 3.4. Dielectric Studies

The trend of conductivity is explained further using a dielectric study. The dialectic constant (*ε_r_*) and dielectric loss (*ε_i_*) are obtained using Equations (11) and (12) [40,41,42]:

(11) $$\varepsilon^{\prime }=\left[\frac{Z^{\prime \prime }}{\omega C_{o}({Z^{\prime }}^{2}+{Z^{\prime \prime }}^{2})}\right]$$

(12) $$\varepsilon^{\prime \prime }=\left[\frac{Z^{\prime }}{\omega C_{o}({Z^{\prime }}^{2}+{Z^{\prime \prime }}^{2})}\right]$$

where *ω* and *C_o_* are the radial frequency and the capacitance in a vacuum, respectively.

**Figure 5:**
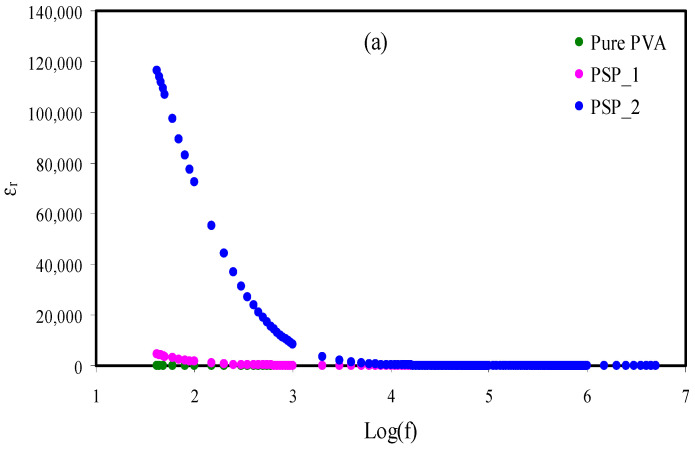

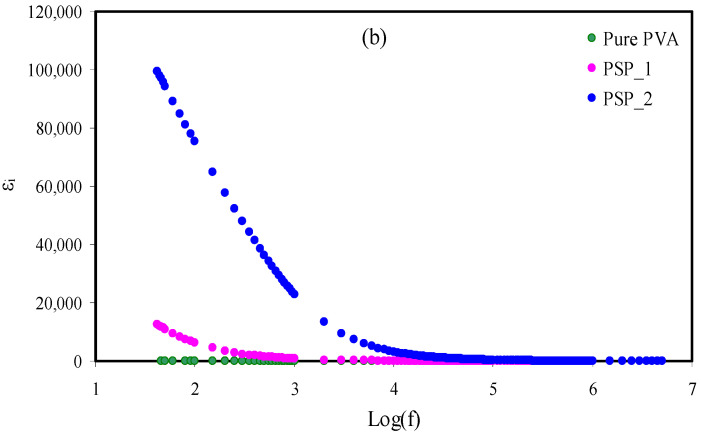
Dielectric plot of (**a**) *ε_r_* v log(f) and (**b**) *ε_i_* v log(f) for pure PVA, PSP_1, and PSP_2 electrolyte systems at RT.

The influence of glycerol on dielectric parameters (*ε_r_* and *ε_i_*) is revealed in Figure 5a,b. The dielectric parameters at the low frequency region increase as the glycerol increases to 40 wt. %. More plasticizer increases the dissociation of ions, and thus provides more mobile ions and increases the conductivity [20].

The agreement between conductivity ($\sigma_{dc}=nq\mu$) and the dielectric constant ($n=n_{o}\exp(-U/\varepsilon_{r}k_{B}T)$) was shown in previous studies [43,44,45,46,47,48,49,50,51,52,53]. In plasticized systems, *ε_r_* and *ε_i_* are low at the high frequency region owing to the swift periodic reversal of the electric field, while both parameters are high at the low frequency region as the blocking electrodes accumulate charges at the electrode/electrolyte interfaces [54,55].

### 3.5. Electrical Modulus Studies

The imaginary and real (*M_i_* and *M_r_*) parts of the complex electrical modulus (M^*^) are measured using Equations (13) and (14) [56,57,58,59,60,61].

(13) $$M^{\prime }=\left[\frac{\varepsilon^{\prime }}{\left({\varepsilon^{\prime }}^{2}+{\varepsilon^{\prime \prime }}^{2}\right)}\right]=\omega C_{o}Z^{\prime \prime }$$

(14) $$M^{\prime \prime }=\left[\frac{\varepsilon^{\prime \prime }}{\left({\varepsilon^{\prime }}^{2}+{\varepsilon^{\prime \prime }}^{2}\right)}\right]=\omega C_{o}Z^{\prime }$$

**Figure 6:**
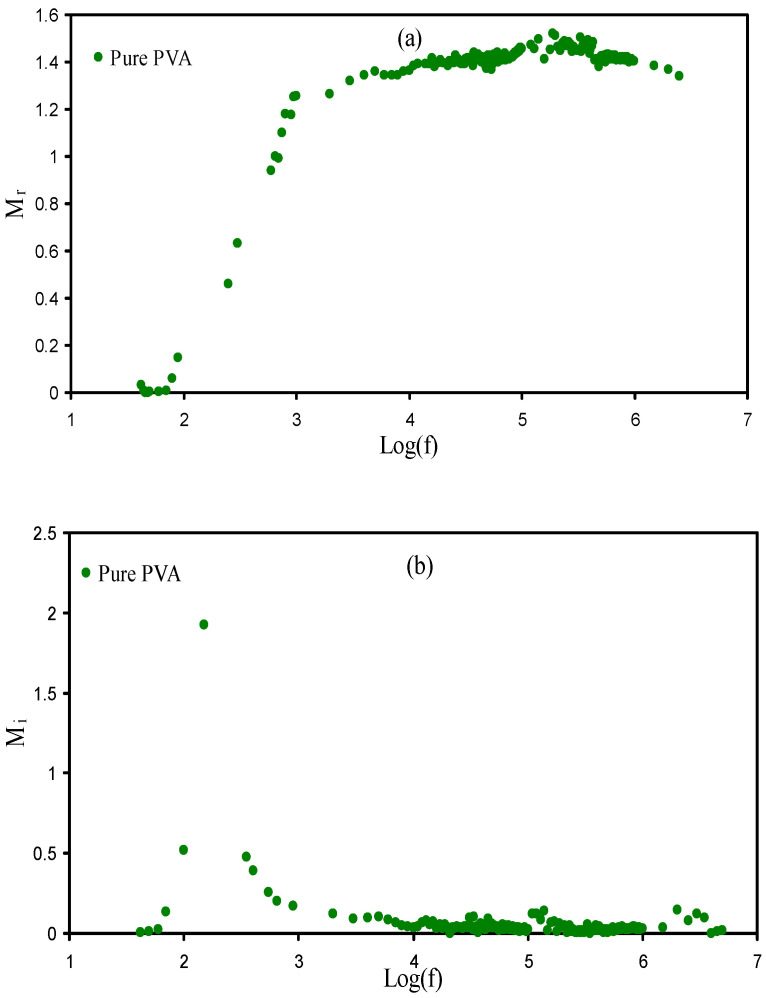
Electric modulus of (**a**) *M_r_* v log(f) and (**b**) *M_i_* v log(f) for pure PVA film at RT.

**Figure 7:**
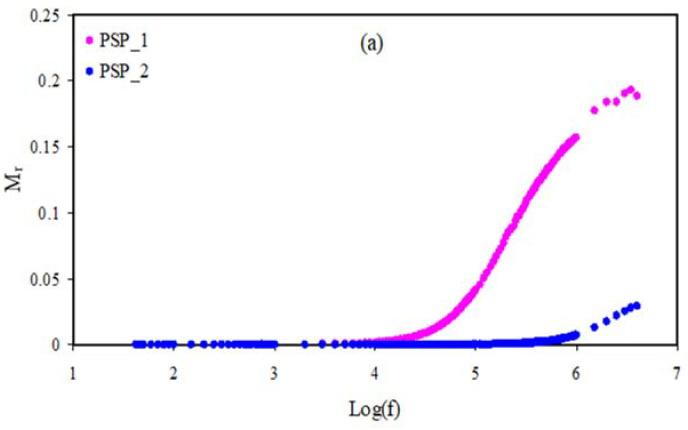

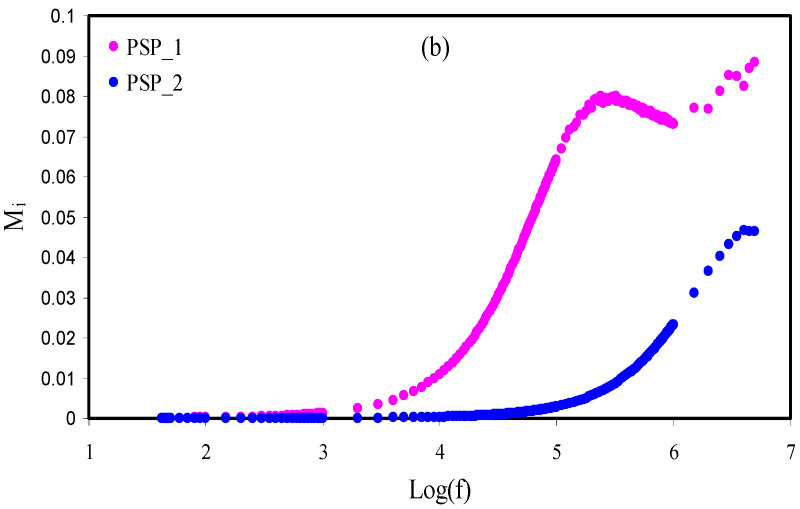
Electric modulus of (**a**) *M_r_* v log(f) and (**b**) *M_i_* v log(f) for PSP_1 and PSP_2 electrolyte samples at RT.

Figure 6a and Figure 7a show *M_r_* against frequency for the pure PVA, PSP_1, and PSP_2 at RT. As seen in the diagram, dispersion is not seen at the low frequency region owing to polarization, and thus the *M_r_* values are close to zero, while dispersion exists at the high frequency region [62]. The long tail at the region of low frequency is associated with the big value of capacitance at the interfaces of the electrodes and electrolytes [63]. Figure 6b and Figure 7b show *M_i_* against frequency for the pure PVA and plasticized systems. In general, the *M_i_* diagram is divided into two regions, which are low and high frequency regions. The low frequency is due to the mechanism of conduction, whereas the high frequency shows the relaxation process [64,65]. It can be seen that in the PSP_1 and PSP_2, the conductivity relaxation peaks appear.

### 3.6. EDLC Characteristics

#### 3.6.1. Transference Number Measurement (TNM)

**Figure 8:**
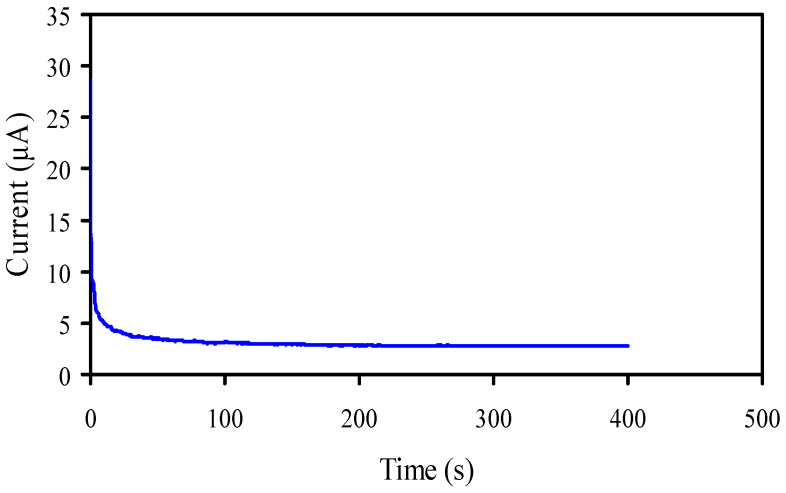
Polarization current vs. time for the highest plasticized sample (PSP_2) at RT.

TNM for the ion (*t_ion_*) and TNM for the electron (*t_e_*) for the PSP_2 sample were measured using the DC polarization method [3]. In this technique, DC voltage is used and the currents are measured against time, as shown in Figure 8. The PSP_2 film was placed between two SS electrodes and the *t_ion_* and *t_e_* were measured using Equations (15) and (16) [3,29,66,67,68,69,70]:

(15) $$t_{ion}=\frac{I_{i}-I_{ss}}{I_{i}}$$

(16) $$t_{el}=1-t_{ion}$$

where steady state and initial current are referred to by *I_i_* and *I_ss_*, respectively.

The cell is polarized when the cell reaches the stationary state, and the transport of the remaining current is only owing to electrons as the SS electrodes block the cations and anions and allow the electrons to pass through it [71]. In the present study, *t_ion_* was 0.902, which is near to an ideal value, meaning that the transport mechanism is dominated by ions in the plasticized films. Shukur and Kadir [72] determined *t_ion_* values are in the range of 0.91 and 0.98 for the NH_4_Cl glycerol-based polymer electrolytes.

From the *t_ion_* and conductivity values, the mobility and diffusion coefficient of anions and cations of each plasticized system were measured using Equations (17)–(22) [73].

(17) $$D=kT\sigma /ne^{2}$$

(18) $$D=D_{+}+D_{-}$$

(19) $$t_{ion}=D_{+}/(D_{+}+D_{-})$$

(20) μ=σ/ne

(21) $$\mu =\mu_{+}+\mu_{-}$$

(22) $$t_{ion}=\mu_{+}/(\mu_{+}+\mu_{-})$$

where *k*, *T*, *σ*, *D___*, *D*_+_, *μ*_+_, and *μ*_−_ are Boltzmann constant, absolute temperature, conductivity, electron charge, anion diffusion coefficient, cation diffusion coefficient, cation mobility, and anion mobility, respectively. The cations are smaller than the anions, and thus the *μ_+_* is higher than the *μ*_−_. Table 4 reveals that the *μ*_+_ and *D*_+_ are higher than the *μ*_−_ and *D*_−_. Once the conductivity is enhanced, the μ_−_ and μ_+_ are increased. The same pattern was observed for *D*_−_ and *D*_+_. So, the TNM shows that the conductivity is impacted by diffusion coefficient and mobility. The PSP_2 has the highest mobility and diffusion coefficient compared with those of the PSP_1 electrolytes.

#### 3.6.2. Electrochemical Stability Study

**Figure 9:**
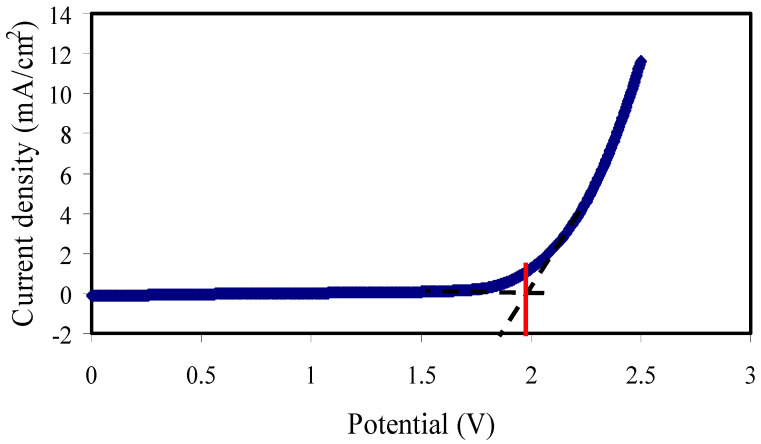
LSV for the highest plasticized sample (PSP_2) at RT.

In order to measure the electrochemical stability window (ESW) of PSP_2, linear sweep voltammetry (LSV) was used. The ESW of the electrolyte is composed of the working voltage limits in which the electrolyte can work safely without decomposing in a device. As shown in Figure 9, the ESW for the SPE film was 1.99 V, which is adequate for the application of the SPE in electrochemical energy storage devices [74,75,76], for example, in a supercapacitor. Hamsan et al. [29] found the ESW of 1.88 V for the system of potato starch/methyl cellulose/NH_4_NO_3_/glycerol and used the electrolyte for an EDLC application.

#### 3.6.3. Cyclic Voltammetry Study

**Figure 10:**
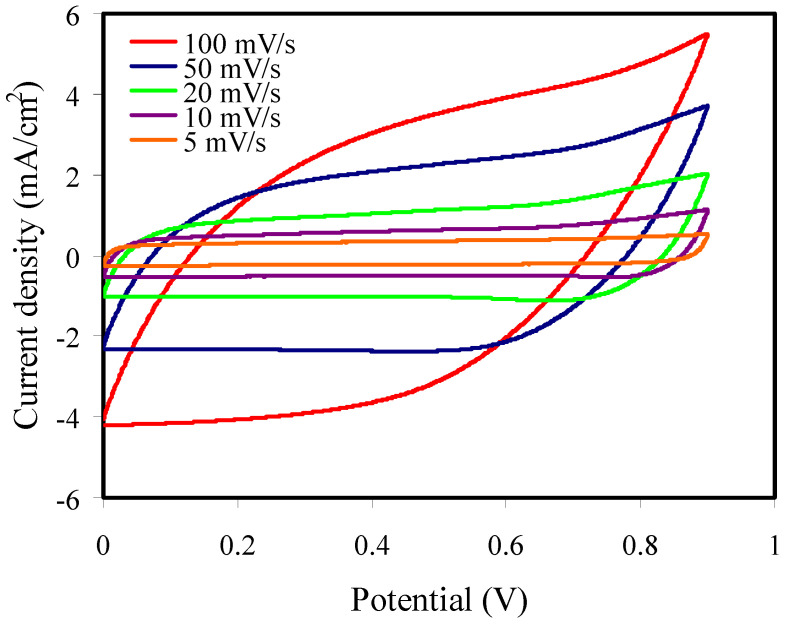
Cyclic voltammetry (CV) curve for the highest plasticized sample (PSP_2) at RT.

The capacitive behavior was examined with the cyclic voltammetry (CV) technique for the cell. Figure 10 indicates the CV response for the EDLC at different scan rates, and the CV curves show a rectangular shape at a lower scan rate. Lack of electron contribution is confirmed by the lack of redox peaks, meaning that the EDLC shows a non-Faradic process [77].

The CV shapes move from a leaf shape to a rectangular one at lower scan rates. The move to a leaf-like shape of the CV curve from a rectangular one is due to carbon porosity and internal resistance, which form current and voltage dependence [78].

The specific capacitance from the CV curve (*C_CV_*) is determined using Equation (23) [33]:

(23) $$C_{CV}=\int_{V_{i}}^{V_{f}}\frac{I\left(V\right)dV}{2ma\left(V_{f}-V_{i}\right)}$$

where *∫I*(*V*)*dV*, *a*, *m*, *V_f,_* and *V_i_* are the area of *CV* curve, scan rate, activate material mass, final (0.9 V), and initial voltages (0 V), respectively.

The values of *C_CV_* are seen in Table 5 in which the *C_CV_* increases at lower scan rates. Ions can fill the vacant sites inside the electrodes as the ions have enough time for the mechanism of diffusion at low scan rates, and thus the *C_CV_* is higher [79]. The *C_CV_* in this work (Table 5) is higher than those of previous works, for example, Hamsan et al. [29] used potato starch/methyl cellulose/NH_4_NO_3_/glycerol for an EDLC and measured a *C_CV_* of 20 F g^−1^ by the CV curve at 2 mV s^−1^. The CV profile in our study is similar to that indicated by Hamsan et al. [29].

### 3.7. Galvanostatic Charge-Discharge (GCD) Analysis

**Figure 11:**
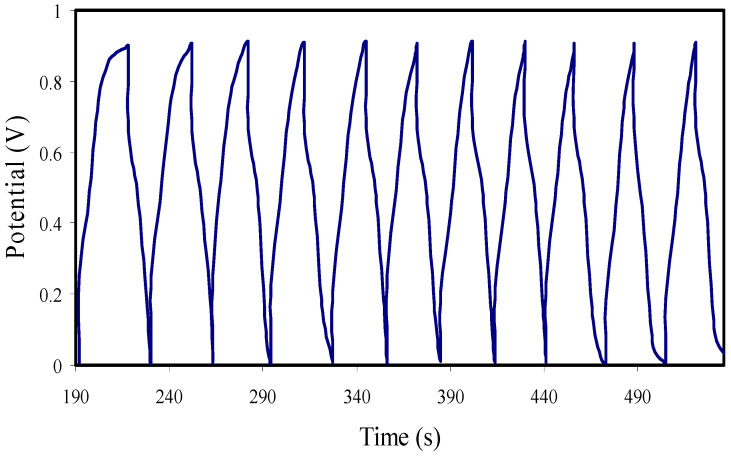
GCD curve at 0.5 mA/cm^2^ for the EDLC at RT.

Figure 11 reveals the GCD profiles at 0.5 mA cm^−2^ with potential from 0 to 1 V. The discharge profiles with nearly linear slope show the capacitive behavior of the EDLC [79], and thus the process of charge storage is based on the development of charges at the electrode/electrolyte interfaces.

**Figure 12:**
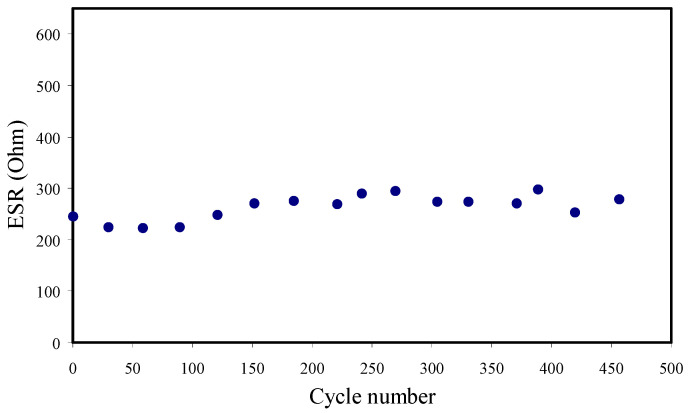
Equivalent series resistance (ESR) of the EDLC device for 450 cycles at RT.

The initial voltage drop in the charge-discharge curves is primarily attributed to the internal resistance of the cell. The equivalent series resistance (ESR) is measured from the voltage drop, and it is presented in Figure 12. For the 450 cycles, ESR ranged from 245 to 278 Ω. A good contact among electrodes and SPEs is ensured by a small ESR, signifying that the journey of cations and anions toward the pore at the electrode’s surface will be easier [80]. Table 6 shows the comparison of the ESR of the EDLC with previous reports.

The *ESR* of the EDLC is measure using Equation (24) [3]:

(24) $$ESR=\frac{V_{d}}{i_{}}$$

where *i* and *V_d_* are the working current and drop voltage before the discharging process, respectively.

**Figure 13:**
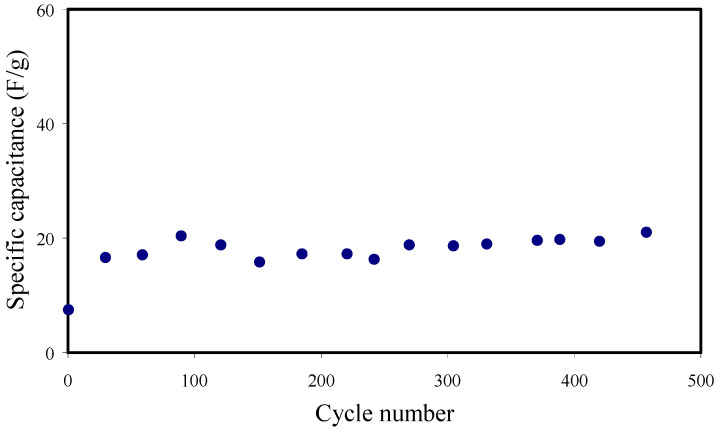
Specific capacitance of the EDLC device for the 450 cycles at RT.

The specific capacitance (*C_d_*) from the galvanostatic charge-discharge (GCD) is measured using Equation (25) [3]:

(25) $$C_{d}=\frac{i}{xm}$$

where *x* and *i* are the discharge gradient and working current, respectively. The *C_CV_* from the CV curve and *C_d_* from GCD are compared. The *C_d_* up to 450 cycles is shown in Figure 13. For the first cycle, the *C_d_* value, measured by Equation (25), is obtained as 7.45 F g^−1^. The *C_d_* value is increased and keeps nearly constant with the average value of ∼18.3 F g^−1^ for 450 cycles beyond the first cycle. This is because most of the cations and anions at the bulk of the electrolyte move gradually in opposite directions toward the surface of the electrodes to create a proper double layer. However, the *C_d_* patterns have slightly dipped after the first cycle, which might be related to the contact between the electrode and electrolyte interfaces and an increase of the internal resistance at higher cycles. The long life cycle with stable capacitance is essential for an EDLC. Table 6 shows the comparison of the *C_d_* of the EDLC with previous reports.

Wei et al. [81] reported that polyethylene oxide (PEO)-based SPEs have large applications in all-solid-state Li-ion batteries. The authors used a simple and effective press-rolling method to decrease the crystallinity of the electrolyte. With the rolled PEO-based SPE, the LiFePO_4_/SPE/Li all-solid Li-ion battery provided a large rechargeable specific capacity of 162.6 mAhg^−1^ with a charge-discharge voltage gap of 60 mV at a current density of 0.2 C with a smaller capacity decay rate. They mentioned that the enhancement of the electrochemical properties was attributed to the method of press-rolling, resulting in a decreased activation energy and also a doubling of conductivity in comparison with those of the electrolyte fabricated by a traditional cast technique. Yu et al. [82] reported that in a fiber-shaped zinc-polyaniline battery (FZPB), the cathode with plasma-treated carbon fibers and a polyaniline loading of 0.158 mg mg_CF_^−1^ (i.e., 2.36 mg cm_CF_^−1^) showed a capacity retention of 95.39% after 200 cycles at 100 mA g^−1^ and a discharge capacity of 83.96 mA h g^−1^ at a large current density of 2000 mA g^−1^, which are ∼1.67 and 1.24 times those of the pure carbon-fiber-based battery, respectively. They also mentioned that the FZPB showed large flexibility with a capacity retention of 86.4% after bending to a radius of 2.5 mm for 100 cycles as a wearable energy device [82].

The other parameters (power density (*P_d_*) and energy density (*E_d_*)) are measured using Equations (26) and (27) [3,29]:

(26) $$E_{d}=\frac{C_{s}V^{2}}{2}$$

(27) $$P_{d}=\frac{V^{2}}{4m(ESR)}$$

where *V* is the applied voltage.

**Figure 14:**
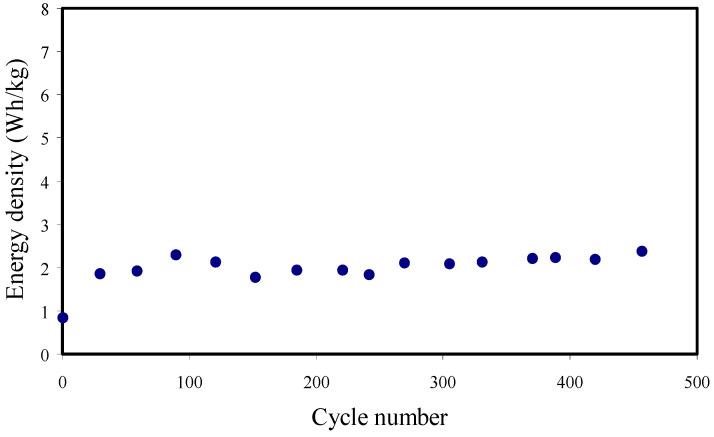
Energy density of the EDLC device for the 450 cycles at RT.

**Figure 15:**
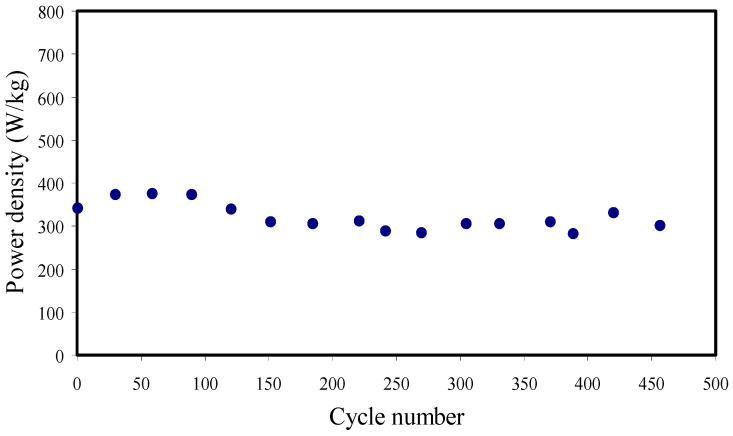
Power density of the EDLC device for the 450 cycles at RT.

From Figure 14, the *E_d_* is 0.84 Wh/kg for the first cycle and becomes more constant at 2.06 Wh/kg beyond the first cycle, meaning that a similar energy barrier is seen by cations and anions when they transport to the surface of the electrodes for the cycles. The *E_d_* has a similar pattern to that of the *C_d_* in Figure 13. The slight decrease in *E_d_* through the cycles is due to the increase of ESR, and thus the energy loss increases through the mechanism of charging-discharging cycles [78,83]. The *P_d_* value measured in this work is shown in Figure 15. The *P_d_* shows a slight drop beyond the 100th cycle within charge-discharge for 450 cycles, and this might be due to the electrolyte depletion. Ion agglomerations after the swift charge and discharge mechanism block the transportation of ions to the electrode surface, and thus the ion adsorption decreases at the electrode-electrolyte interfaces [84]. Table 6 indicates the comparison of the *P_d_* and *E_d_* of the EDLC with those of previous reports. Shim et al. [85] developed a dual anion-doped PVA gel electrolyte with Methanesulfonic acid (MSA) as a multi-functional additive to improve the electrochemical performance and wearability of quasi-solid-state fiber-shaped Zn-polyaniline batteries (Fs-ZPBs). The MSA-based Fs-ZPB revealed a great rate capability, showing long cycling stability (capacity retention = 88.1% after 2000 cycles), a specific capacity of 100.3 mAh g^−1^ at 5 A g^−1^, and high power and energy densities of 9135.4 W kg^−1^ and 115.4 Wh kg^−1^, respectively. Furthermore, the prepared Fs-ZPBs showed high flexibility with a capacity retention of 92.7% after 500 cycles at a bending radius of 2.5 mm [85].

## 4. Conclusions

PVA/NH_4_SCN/glycerol-based electrolytes were formed by the solution casting method. Using FTIR, the interaction of the elements of the electrolyte was established at 40 wt. % glycerol. The 40 wt. % glycerol addition increased the DC conductivity to 1.82 × 10^−5^ S cm^−1^. It was shown that when the glycerol increased, the number density (*n*), mobility (*μ*), and diffusion coefficient (*D*) of the ions improved. It was shown by the FESEM that the PSP_2 has a smooth surface morphology. The conductivity trend was further verified by dielectric examination. TNM showed that the majority of carriers were cations and anions, and the t_ion_ and t_e_ for the PSP_2 were measured to be 0.902 and 0.097, respectively. LSV showed that the SEW for the PSP_2 was at 1.99 V, showing the eligibility of the SPE in the EDLC application. The profile of CV indicates a rectangle with the absence of redox peaks, which confirms the ELDC capacitive behavior. The C_d_, E_d_, and P_d_ obtained for the EDLC were 18.3 F g^−1^, 2.06 Wh/kg, and 318.73 W/kg, respectively. The ESR of the EDLC throughout 450 cycles varied from 245 to 278 Ω.

## Figures and Tables

**Table 1:** The FTIR results of PVA and plasticized systems.

Assignments	Wavenumber (cm^−1^)		
	PVA	PSP_1	PSP_2
O–H stretching	3303	3305	3309
C–H stretching	2902	2914	2909
Aromatic S–C = *n* stretching	-	2043	2042
C=O stretching	1638	1609	1605
C–H bending of CH_2_	1412	1407	1413
C–H deformation	1318	1310	1309
–C–O– stretching	1080	1029	1038
C–H rocking	833	831	822

**Table 2:** The EEC fitting parameters for PVA, PSP_1, and PSP_2 at RT.

Sample	P_1_ (rad)	P_2_ (rad)	*K*_1_ (F^−1^)	*K*_2_ (F^−1^)	*C*_1_ (F)	*C*_2_ (F)
PVA	0.79	-	3.6 × 10^10^	-	2.78 × 10^−11^	-
PSP_1	0.86	0.40	2.2 × 10^9^	1.6 × 10^5^	4.55 × 10^−10^	6.25 × 10^−6^
PSP_2	0.87	0.69	3.8 × 10^8^	1.09 × 10^5^	2.63 × 10^−9^	9.17 × 10^−6^

**Table 3:** The conductivity of the films at RT.

Symbol	PVA (wt. %)/NH_4_SCN (wt. %)/Glycerol (wt. %)	*σ_dc_* (S cm^−1^)
Pure PVA	50:00:00	2.87 × 10^−11^
PSP_1	50:50:30	5.45 × 10^−7^
PSP_2	50:50:40	1.82 × 10^−5^

**Table 4:** The transport parameters of ions at RT.

Sample	*D* (cm^2^ s^−1^)	µ (cm^2^ V^−1^s)	*n* (cm^−3^)	*D*_+_ (cm^2^ s^−1^)	D_−_ (cm^2^ s^−1^)	µ_+_ (cm^2^ V^−1^s)	µ_−_ (cm^2^ V^−1^s)
PSP_1	7.27 × 10^−9^	2.83 × 10^−7^	1.20 × 10^19^	6.55 × 10^−9^	7.12 × 10^−10^	2.55 × 10^−7^	2.77 × 10^−8^
PSP_2	2.59 × 10^−8^	1.01 × 10^−6^	1.12 × 10^20^	2.34 × 10^−8^	2.54 × 10^−9^	9.11 × 10^−7^	9.9 × 10^−8^

**Table 5:** Capacitance from CV curves.

Scan Rate (mVs^−1^)	C_CV_ (F g^−1^)
100	19.43
50	29.26
20	40.00
10	44.58
5	45.72

**Table 6:** EDLC parameters using different polymer electrolytes at RT.

Electrolyte Systems	Specific Capacitance (F/g)	Energy Density (Wh/kg)	Power Density (W/kg)	ESR (Ω)	Cycle Number	Ref.
Chitosan/methyl cellulose (MC)/NH_4_I/glycerol	9.70	1.1	578.55	136–155	100	[86]
Chitosan-κ-carrageenan/NH_4_NO_3_	18.5	-	-	-	20	[13]
MC/NH_4_NO_3_/poly(ethylene glycol)	25	2.5	130	109	-	[28]
PVA/chitosan/NH_4_I/glycerol	19.4	5.48	380	75–330	250	[87]
MC/potato starch (PS)/NH_4_NO_3_/glycerol	31	3.1	910–385	29–64	1000	[29]
Chitosan/PS/NH_4_F	4.1	0.57	155	550	100	[88]
PVA/NH_4_SCN/glycerol	18.3	2.06	318.73	245–278	450	This work

## Data Availability

Not applicable.

## References

[1] Burk A. (2000). Ultracapacitors: Why, how, and where is the technology. J. Power Sources.

[2] Kötz R., Carlen M. (2000). Principles and applications of electrochemical capacitors. Electrochim. Acta.

[3] Brza M.A., Aziz S.B., Anuar H., Ali F. (2020). Structural, Ion Transport Parameter and Electrochemical Properties of Plasticized Polymer Composite Electrolyte Based on PVA: A Novel Approach to Fabricate High Performance EDLC Devices. Polym. Test..

[4] Song J.Y., Wang Y.Y., Wan C.C. (1999). Review of gel-type polymer electrolytes for lithium-ion batteries. J. Power Sources.

[5] Groce F., Gerace F., Dautzemberg G., Passerini S., Appetecchi G.B., Scrosati B. (1994). Synthesis and characterization of highly conducting gel electrolytes. Electrochim. Acta.

[6] Pistoia G., Antonini A., Wang G. (1996). Impedance study on the reactivity of gel polymer electrolytes towards a lithium electrode. J. Power Sources.

[7] Tsunemi K., Ohno H., Tsuchida E. (1983). Conduction of lithium ions in polyvinylidene fluoride and its derivatives-I. Electrochim. Acta.

[8] Magistris A., Quartarone E., Mustarelli P., Saito Y., Kataoka H. (2002). PVDF-based porous polymer electrolytes for lithium batteries. Solid State Ion..

[9] Hemalatha R., Alagar M., Selvasekarapandian S., Sundaresan B., Moniha V. (2019). Studies of proton conducting polymer electrolyte based on PVA, amino acid proline and NH_4_SCN. J. Sci. Adv. Mater. Devices.

[10] Hema M., Selvasekarapandian S., Arunkumar D., Sakunthala A., Nithya H. (2009). FTIR, XRD and ac impedance spectroscopic study on PVA based polymer electrolyte doped with NH_4_X (X = Cl, Br, I). J. Non-Cryst. Solids.

[11] Asnawi A.S.F.M., Aziz S.B., Brevik I., Brza M.A., Yusof Y.M., Alshehri S.M., Ahamad T., Kadir M.F.Z. (2021). The study of plasticized sodium ion conducting polymer blend electrolyte membranes based on chitosan/dextran biopolymers: Ion transport, structural, morphological and potential stability. Polymers.

[12] Dey A., Karan S., Dey A., De S.K. (2011). Structure, morphology and ionic conductivity of solid polymer electrolyte. Mater. Res. Bull..

[13] Shuhaimi N.E.A., Alias N.A., Majid S.R., Arof A.K. (2008). Electrical double layer capacitor with proton conducting κ-carrageenanchitosan electrolytes. Funct. Mater. Lett..

[14] Srivastava N., Chandra A., Chandra S. (1995). Dense branched growth of (SCN)x and ion transport in the poly(ethyleneoxide) NH_4_SCN polymer electrolyte. Phys. Rev. B.

[15] Pawlicka A., Danczuk M., Wieczorek W., Zygadło-Monikowska E. (2008). Influence of plasticizer type on the properties of polymer electrolytes based on chitosan. J. Phys. Chem. A.

[16] Liu G., Kim J.Y., Wang M., Woo J.Y., Wang L., Zou D., Lee J.K. (2018). Soft, Highly Elastic, and Discharge-Current-Controllable Eutectic Gallium–Indium Liquid Metal–Air Battery Operated at Room Temperature. Adv. Energy Mater..

[17] Makled M.H., Sheha E., Shanap T.S., El-Mansy M.K. (2013). Electrical conduction and dielectric relaxation in p-type PVA/CuI polymer composite. J. Adv. Res..

[18] Hema M., Selvasekerapandian S., Sakunthala A., Arunkumar D., Nithya H. (2008). Structural, vibrational and electrical characterization of PVA-NH_4_Br polymer electrolyte system. Phys. B Condens. Matter.

[19] Noor N.A.M., Isa M.I.N. (2015). Structural and Conduction Studies of Solid Biopolymer Electrolytes System Based on Carboxymethyl Cellulose. Am. Eurasian J. Sustain. Agric..

[20] Liang S., Huang Q., Liu L., Yam K.L. (2009). Microstructure and molecular interaction in glycerol plasticized chitosan/poly(vinyl alcohol) blending films. Macromol. Chem. Phys..

[21] Kharazm A.N., Faraji R.M., Hussin E., Saion W.M.M., Yunus M., Behazad K. (2015). Structural, optical, opto-thermal and thermal properties of ZnS–PVA nanofluids synthesized through a radiolytic approach. Beilstein J. Nanotechnol..

[22] Jiang L., Yang T., Peng L., Dan Y. (2015). Acrylamide modified poly(vinyl alcohol): Crystalline and enhanced water solubility. RSC Adv..

[23] Selvasekarapandian S., Baskaran R., Hema M. (2005). Complex AC impedance, transference number and vibrational spectroscopy studies of proton conducting PVAc–NH_4_SCN polymer electrolytes. Phys. B.

[24] Teo L.P., Buraidah M.H., Nor A.F.M., Majid S.R. (2012). Conductivity and dielectric studies of Li_2_SnO_3_. Ionics.

[25] Samsudin A.S., Khairul W.M., Isa M.I.N. (2012). Characterization on the potential of carboxy methylcellulose for application as proton conducting biopolymer electrolytes. J. Non Cryst. Solids.

[26] Malathi J., Kumaravadivel M., Brahmanandhan G.M., Hema M., Baskaran R., Selvasekarapandian S. (2010). Structural, thermal and electrical properties of PVALiCF_3_SO_3_ polymer electrolyte. J. Non-Cryst. Solids.

[27] Qian X., Gu N., Cheng Z., Yang X., Wang E., Dong S. (2001). Impedance study of (PEO)10LiClO_4_–Al_2_O_3_ composite polymer electrolyte with blocking electrodes. Electrochim. Acta.

[28] Shuhaimi N.E.A., Teo L.P., Woo H.J., Majid S.R., Arof A.K. (2012). Electrical double-layer capacitors with plasticized polymer electrolyte based on methyl cellulose. Polym. Bull..

[29] Hamsan M.H., Shukur M.F., Kadir M.F.Z. (2017). NH_4_NO_3_ as charge carrier contributor in glycrolized potato starch-methyl cellulose blend-based polymer electrolyte and the application in electrochemical double-layer capacitor. Ionics.

[30] Aziz S.B., Abdullah R.M. (2018). Crystalline and amorphous phase identification from the tanδ relaxation peaks and impedance plots in polymer blend electrolytes based on [CS: AgNt ]x: PEO (x-1) (10 ≤ x ≤ 50). Electrochim. Acta.

[31] Dannoun E.M.A., Aziz S.B., Brza M.A., Nofal M.M., Asnawi A.S.F.M., Yusof Y.M., Al-Zangana S., Hamsan M.H., Kadir M.F.Z., Woo H.J. (2020). The Study of Plasticized Solid Polymer Blend Electrolytes Based on Natural Polymers and Their Application for Energy Storage EDLC Devices. Polymers.

[32] Aziz S.B., Brza M.A., Dannoun E.M.A., Hamsan M.H., Hadi J.M., Kadir M.F.Z., Abdulwahid R.T. (2020). The study of electrical and electrochemical properties of magnesium ion conducting CS: PVA based polymer blend electrolytes: Role of lattice energy of magnesium salts on EDLC performance. Molecules.

[33] Mustafa M.S., Ghareeb H.O., Aziz S.B., Brza M.A., Al-Zangana S., Hadi J.M., Kadir M.F.Z. (2020). Electrochemical Characteristics of Glycerolized PEO-Based Polymer Electrolytes. Membranes.

[34] Hamsan M.H., Shukur M.F., Kadir M.F.Z. (2016). The effect of NH_4_NO_3_ towards the conductivity enhancement and electrical behavior in methyl cellulose-starch blend based ionic conductors. Ionics.

[35] Gondaliya N., Kanchan D.K., Sharma P. (2013). Effect of a plasticizer on a solid polymer electrolyte. Soc. Plast. Eng..

[36] Arof A.K., Amirudin S., Yusof S.Z., Noor I.M. (2014). A method based on impedance spectroscopy to determine transport properties of polymer electrolytes. Phys. Chem. Chem. Phys..

[37] Shukur M.F., Ithnin R., Kadir M.F.Z. (2014). Electrical properties of proton conducting solid biopolymer electrolytes based on starch–chitosan blend. Ionics.

[38] YYusof M., Shukur M.F., Hamsan M.H., Jumbri K., Kadir M.F.Z. (2019). Plasticized solid polymer electrolyte based on natural polymer blend incorporated with lithium perchlorate for electrical double-layer capacitor fabrication. Ionics.

[39] Mobarak N.N., Ahmad A., Abdullah M.P., Ramli N., Rahman M.Y.A. (2013). Conductivity enhancement via chemical modification of chitosan based green polymer electrolyte. Electrochim. Acta.

[40] Hamsan H.M., Aziz S.B., Kadir M.F.Z., Brza M.A., Karim W.O. (2020). The study of EDLC device fabricated from plasticized magnesium ion conducting chitosan based polymer electrolyte. Polym. Test..

[41] Woo H.J., Majid S.R., Arof A.K. (2012). Dielectric properties and morphology of polymer electrolyte based on poly(ɛ-caprolactone) and ammonium thiocyanate. Mater. Chem. Phys..

[42] Hamsan M.H., Shukur M.F., Aziz S.B., Kadir M.F.Z. (2019). Dextran from Leuconostoc mesenteroides-doped ammonium salt-based green polymer electrolyte. Bull. Mater. Sci..

[43] Khiar A.S., Arof A.K. (2010). Conductivity studies of starch-based polymer electrolytes. Ionics.

[44] Aziz S.B. (2015). Study of electrical percolation phenomenon from the dielectric and electric modulus analysis. Bull. Mater. Sci..

[45] Aziz S.B., Abidin Z.H.Z., Arof A.K. (2010). Influence of silver ion reduction on electrical modulus parameters of solid polymer electrolyte based on chitosan-silver triflate electrolyte membrane. Express Polym. Lett..

[46] Aziz S.B., Kadir M.F.Z., Abidin Z.H.Z. (2016). Structural, morphological and electrochemical impedance study of CS: LiTf based solid polymer electrolyte: Reformulated arrhenius equation for ion transport study. Int. J. Electrochem. Sci..

[47] Aziz N.A., Majid S.R., Arof A.K. (2012). Synthesis and characterizations of phthaloyl chitosan-based polymer electrolytes. J. Non. Cryst. Solids.

[48] Aziz S.B., Woo T.J., Kadir M.F.Z., Ahmed H.M. (2018). A conceptual review on polymer electrolytes and ion transport models. J. Sci. Adv. Mater. Devices.

[49] Aziz S.B., Abidin Z.H.Z. (2015). Ion-transport study in nanocomposite solid polymer electrolytes based on chitosan: Electrical and dielectric analysis. J. Appl. Polym. Sci..

[50] Aziz S.B. (2013). Li^+^ ion conduction mechanism in poly (ε-caprolactone)-based polymer electrolyte. Iran. Polym. J..

[51] Aziz S.B., Abidin Z.H.Z. (2014). Electrical and morphological analysis of chitosan:AgTf solid electrolyte. Mater. Chem. Phys..

[52] Aziz S.B., Abdullah R.M., Rasheed M.A., Ahmed H.M. (2017). Role of ion dissociation on DC conductivity and silver nanoparticle formation in PVA:AgNt based polymer electrolytes: Deep insights to ion transport mechanism. Polymers.

[53] Shukur M.F., Ibrahim F.M., Majid N.A., Ithnin R., Kadir M.F.Z. (2013). Electrical analysis of amorphous corn starch-based polymer electrolyte membranes doped with LiI. Phys. Scr..

[54] Selvasekarapandian S., Chithra D.R. (1999). Dielectric studies on a solid electrolyte AgI-PbBr_2_-Ag_2_O-B_2_O_3_. Mater. Chem. Phys..

[55] Iqbal M.Z., Rafiuddin S.R. (2016). Structural, electrical conductivity and dielectric behavior of Na_2_SO_4_–LDT composite solid electrolyte. J. Adv. Res..

[56] Aziz S.B., Marf A.S., Dannoun E.M.A., Brza M.A., Abdullah R.M. (2020). The Study of the Degree of Crystallinity, Electrical Equivalent Circuit, and Dielectric Properties of Polyvinyl Alcohol (PVA)-Based Biopolymer Electrolytes. Polymers.

[57] Hadi J.M., Aziz S.B., Mustafa M.S., Brza M.A., Hamsan M.H., Kadir M.F.Z., Ghareeb H.O., Hussein S.A. (2020). Electrochemical impedance study of proton conducting polymer electrolytes based on PVC doped with thiocyanate and plasticized with glycerol. Int. J. Electrochem. Sci..

[58] Aziz S.B., Abdullah R.M., Kadir M.F.Z., Ahmed H.M. (2019). Non suitability of silver ion conducting polymer electrolytes based on chitosan mediated by barium titanate (BaTiO_3_) for electrochemical device applications. Electrochim. Acta.

[59] Aziz S.B. (2016). Occurrence of electrical percolation threshold and observation of phase transition in chitosan (1 − x): AgI x (0.05 ≤ x ≤ 0.2)-based ion-conducting solid polymer composites. Appl. Phys. A Mater. Sci. Process..

[60] Aziz S.B. (2018). The mixed contribution of ionic and electronic carriers to conductivity in chitosan based solid electrolytes mediated by CuNt salt. J. Inorg. Organomet. Polym. Mater..

[61] Aziz S.B., Brza M.A., Saed S.R., Hamsan M.H., Kadir M.F.Z. (2020). Ion association as a main shortcoming in polymer blend electrolytes based on CS: PS incorporated with various amounts of ammonium tetrafluoroborate. J. Mater. Res. Technol..

[62] Gurusiddappa J., Madhuri W., Suvarna R.P., Dasan K.P. (2016). Conductivity and dielectric behavior of polyethylene oxidelithium perchlorate solid polymer electrolyte films. Indian J. Adv. Chem. Sci..

[63] Khiar A.S.A., Puteh R., Arof A.K. (2006). Conductivity studies of a chitosan-based polymer electrolyte. Phys. B.

[64] Aziz S.B., Karim W.O., Ghareeb H.O. (2020). The deficiency of chitosan: AgNO_3_ polymer electrolyte incorporated with titanium dioxide filler for device fabrication and membrane separation technology. J. Mater. Res. Technol..

[65] Hadi J.M., Aziz S.B., Mustafa M.S., Hamsan M.H., Abdulwahid R.T., Kadir M.F.Z., Ghareeb H.O. (2020). Role of nano-capacitor on dielectric constant enhancement in PEO:NH_4_SCN:xCeO2 polymer nano-composites: Electrical and electrochemical properties. J. Mater. Res. Technol..

[66] Hadi J.M., Aziz S.B., Saeed S.R., Brza M.A., Abdulwahid R.T., Hamsan M.H., Abdullah R.M., Kadir M.F.Z., Muzakir S.K. (2020). Investigation of ion transport parameters and electrochemical performance of plasticized biocompatible chitosan-based proton conducting polymer composite electrolytes. Membranes.

[67] Aziz S.B., Hamsan M.H., Abdullah R.M., Kadir M.F.Z. (2019). A promising polymer blend electrolyte based on chitosan: Methyl cellulose for EDLC application with high specific capacitance and energy density. Molecules.

[68] Hadi J.M., Aziz S.B., Nofal M.M., Hussen S.A., Hamsan M.H., Brza M.A., Abdulwahid R.T., Kadir M.F.Z., Woo H.J. (2020). Electrical, dielectric property and electrochemical performances of plasticized silver ion-conducting chitosan-based polymer nanocomposites. Membranes.

[69] Hamsan M.H., Aziz S.B., Nofal M.M., Brza M.A., Abdulwahid R.T., Hadi J.M., Karim W.O., Kadir M.F.Z. (2020). Characteristics of EDLC device fabricated from plasticized chitosan: MgCl_2_ based polymer electrolyte. J. Mater. Res. Technol..

[70] Kobayashi T., Noguchi Y., Miyayama M. (2005). Enhanced spontaneous polarization in superlattice structure Bi_4_Ti_3_O_12_-BaBi_4_Ti_4_O_15_ single crystal. Appl. Phys. Lett..

[71] Rani M.S.A., Ahmad A., Mohamed N.S. (2017). Influence of nano-sized fumed silica on physicochemical and electrochemical properties of cellulose derivatives-ionic liquid biopolymer electrolytes. Ionics.

[72] Shukur M.F., Kadir M.F.Z. (2015). Hydrogen ion conducting starch-chitosan blend based electrolyte for application in electrochemical devices. Electrochim. Acta.

[73] Aziz S.B., Hadi J.M., Elham E.M., Abdulwahid R.T., Saeed S.R., Marf A.S., Karim W.O., Kadir M.F.Z. (2020). The study of plasticized amorphous biopolymer blend electrolytes based on polyvinyl alcohol (PVA): Chitosan with high ion conductivity for energy storage electrical double-layer capacitors (EDLC) device application. Polymers.

[74] Azli A.A., Manan N.S.A., Aziz S.B., Kadir M.F.Z. (2020). Structural, impedance and electrochemical double-layer capacitor characteristics of improved number density of charge carrier electrolytes employing potato starch blend polymers. Ionics.

[75] Asnawi A.S.F.M., Aziz S.B., Saeed S.R., Yusof Y.M., Abdulwahid R.T., Al-Zangana S., Karim W.O., Kadir M.F.Z. (2020). Solid-State EDLC Device Based on Magnesium Ion-Conducting Biopolymer Composite Membrane Electrolytes: Impedance, Circuit Modeling, Dielectric Properties and Electrochemical Characteristics. Membranes.

[76] Moniha V., Alagar M., Selvasekarapandian S., Sundaresan B., Hemalatha R., Boopathi G. (2018). Synthesis and characterization of bio-polymer electrolyte based on iota-carrageenan with ammonium thiocyanate and its applications. J. Solid State Electrochem..

[77] Hashmi S.A., Latham R.J., Linford R.G., Schilndwein S.W. (1997). Polymer electrolyte based solid state redox supercapacitors withpoly(3-methyl thiophene) and polypyrrole conducting polymer electrodes. Ionics.

[78] Kadir M.F.Z., Arof A.K. (2011). Application of PVA–chitosan blend polymer electrolyte membrane in electrical double layer capacitor. Materials Research Innovations. Mater. Res. Innov..

[79] Teoh K.H., Liew C.W., Ramesh S. (2014). Electric double layer capacitor based on activated carbon electrode and biodegradable composite polymer electrolyte. Ionics.

[80] Asmara S.N., Kufian M.Z., Majid S.R., Arof A.K. (2011). Preparation and characterization of magnesium ion gel polymer electrolytes for application in electrical double layer capacitors. Electrochim. Acta.

[81] Wei Z., Ren Y., Wang M., He J., Huo W., Tang H. (2020). Improving the Conductivity of Solid Polymer Electrolyte by Grain Reforming. Nanoscale Res. Lett..

[82] Yu H., Liu G., Wang M., Ren R., Shim G., Kim J.Y., Tran M.X., Byun D., Lee J.K. (2020). Plasma-Assisted Surface Modification on the Electrode Interface for Flexible Fiber-Shaped Zn-Polyaniline Batteries. ACS Appl. Mater. Interfaces.

[83] Wei Y.Z., Fang B., Iwasa S., Kumagai M. (2005). A novel electrode material for electric double-layer capacitors. J. Power Source.

[84] Liew C.W., Ramesh S., Arof A.K. (2016). Enhanced capacitance of EDLCs (electrical double layer capacitors) based on ionic liquid added polymer electrolytes. Energy.

[85] Shim G., Tran X.M., Liu G., Byun D., Lee K.J. (2021). Flexible, fiber-shaped, quasi-solid-state Zn-polyaniline batteries with methanesulfonic acid-doped aqueous gel electrolyte. Energy Storage Mater..

[86] Aziz S.B., Hamsan M.H., Brza M.A., Kadir M.F.Z., Muzakir S.K., Abdulwahid R.T. (2020). Effect of glycerol on EDLC characteristics of chitosan: Methylcellulose polymer blend electrolytes. J. Mater. Res. Technol..

[87] Marf A.S., Aziz S.B., Abdullah R.M. (2020). Plasticized H^+^ ion-conducting PVA:CS-based polymer blend electrolytes for energy storage EDLC application. J. Mater. Sci. Mater. Electron..

[88] Aziz S.B., Hamsan M.H., Karim W.O., Marif A.S., Abdulwahid R.T., Kadir M.F.Z., Brza M.A. (2020). Study of impedance and solid-state double-layer capacitor behavior of proton (H^+^)-conducting polymer blend electrolyte-based CS:PS polymers. Ionics.

